# Reactive Oxygen and Nitrogen Species in Myocardial Infarction: Mechanistic Insights and Clinical Correlations

**DOI:** 10.3390/medsci13030152

**Published:** 2025-08-24

**Authors:** Hussein M. Ismail, Sameh A. Ahmed, Ahmed M. Alsaedi, Waleed H. Almaramhy, Man K. Alraddadi, Muhannad S. Albadrani, Ibraheam M. Alhejaily, Faisal A. Mohammad, Anas M. Ghaith, Ali A. Youssef

**Affiliations:** 1 Department of Internal Medicine, College of Medicine, Taibah University, Al-Madinah Al-Munawarah 30001, Saudi Arabia; 2 Department of Cardiology, College of Medicine, Suez Canal University, Ismailia 41522, Egypt; 3 Department of Pharmacognosy and Pharmaceutical Chemistry, College of Pharmacy, Taibah University, Al-Madinah Al-Munawarah 30001, Saudi Arabia

**Keywords:** myocardial infarction, coronary artery disease, oxidative stress, reactive oxygen species, reactive nitrogen species

## Abstract

Background/Objectives: Myocardial infarction (MI) remains a leading cause of morbidity and mortality worldwide, driven largely by underlying coronary artery disease (CAD). Reactive oxygen species (ROS) and reactive nitrogen species (RNS) play pivotal mechanistic roles in endothelial dysfunction, atherosclerotic plaque progression, and subsequent cardiac injury. Excessive production of these reactive species disrupts cellular redox balance, promotes mitochondrial dysfunction, and accelerates vascular inflammation, ultimately contributing to plaque rupture and MI. This study aimed to investigate the mechanistic associations and clinical correlations of individual ROS and RNS markers in patients with MI. Methods: We conducted a case–control study including 86 patients with MI and 60 age- and sex-matched controls without cardiovascular disease, recruited from the Medina Cardiac Center in Saudi Arabia. The MI cohort was subdivided into ST-elevation MI (STEMI, *n* = 62) and non-ST-elevation MI (NSTEMI, *n* = 24) to explore potential differences in oxidative and nitrosative stress profiles. Serum levels of multiple ROS (including hydrogen peroxide, hydroxyl radical, and superoxide anion) and RNS (including nitric oxide and peroxynitrite) were quantified using validated fluorescence-based assays. Clinical and biochemical parameters, including lipid profiles, troponin, and left ventricular ejection fraction, were also assessed. Results: Most ROS and RNS markers were significantly elevated in MI patients compared to controls (*p* < 0.05), except for nitrogen dioxide. Moderate to strong positive correlations were observed between ROS/RNS levels and serum total cholesterol and LDL-cholesterol (*p* < 0.001). In contrast, weak or non-significant correlations were found between ROS/RNS markers and serum troponin or left ventricular ejection fraction. Both STEMI and NSTEMI subgroups demonstrated significantly higher oxidative and nitrosative stress levels compared to controls, with distinct patterns between the subtypes. Conclusions: This study underscores a mechanistic link between elevated ROS/RNS levels and myocardial infarction, supporting the importance of targeting oxidative and nitrosative pathways as potential therapeutic strategies.

## 1. Introduction

Coronary artery disease (CAD) represents a significant health burden; despite advancements in management and primary prevention, the mortality rate remains relatively high in Saudi Arabia (42%), with an estimated CAD prevalence of 5.5% [1,2]. The high incidence of heart attacks, which account for 19% of all deaths in Saudi Arabia, underscores the necessity of effective prevention, early detection, and treatment strategies [3].

ROS and RNS play a crucial role in myocardial infarction (MI) and its pathophysiology. Oxidative stress and cardiomyocyte damage occur due to increased ROS and RNS production during MI [4]. ROS in cardiomyocytes originate from various sources such as the mitochondrial electron transport chain, NADPH oxidase, nitric oxide synthase, xanthine oxidase, and lipoxygenase/cyclooxygenase. ROS and RNS impact the survival, proliferation, and differentiation of cardiac progenitor cells, which are essential for myocardial homeostasis [5,6].

Reactive oxygen species are small molecules derived from oxygen and produced during normal cellular metabolism. Reactive nitrogen species (RNS) are nitrogen-based oxidants that often contain oxygen, making them closely related to ROS. Under physiological conditions, ROS function as signaling molecules. However, when ROS levels exceed antioxidant capacity, oxidative stress ensues, altering cellular function and contributing to disease progression [7]. ROS are produced by all vascular layers, including the endothelium and smooth muscle cells [8]. They play a key role in vascular homeostasis by acting as secondary messengers in signaling pathways that regulate vascular smooth muscle cell function, endothelial cell proliferation, and platelet activation. Furthermore, ROS contributes to platelet activation, which influences vascular balance [9].

ROS and RNS are classified into two groups: radical and non-radical species. Radical ROS include hydroxyl radical (•OH), superoxide anion (O_2_•^−^), hypochlorite radical (OHCl•), peroxyl radical (ROO•), nitric oxide (NO), and nitrogen dioxide (ONO-), whereas non-radical ROS include hydrogen peroxide (H_2_O_2_), singlet oxygen (^1^O_2_), and peroxynitrite (ONOO-) [10].

Oxidative stress results from an imbalance between ROS/RNS production and antioxidant defense mechanisms. During oxidative phosphorylation, 1–3% of oxygen is converted into superoxide anions in mitochondrial complexes I and III. Due to the high mitochondrial density in cardiac myocytes, myocardial oxygen consumption is high, increasing oxidative stress and contributing to atherosclerosis [11,12]. Additionally, NADH/NADPH oxidase (NOX) becomes active on cell membranes during phagocytosis, leading to increased ROS generation [13].

Excessive ROS production causes oxidative stress in coronary arteries, contributing to CAD development. ROS play a pivotal role in atherosclerosis by oxidizing LDL, activating metalloproteinases, and promoting plaque rupture and MI [8,14].

The use of antioxidants and enzyme-based therapies for managing MI and promoting post-MI recovery has been thoroughly investigated in scientific studies [15,16,17]. Medications targeting oxidative stress, such as spin traps and flavonoids that function by removing ROS and reducing oxidative damage to cardiac cells, are a crucial step in the early phases of a heart attack. For instance, flavonoids have been demonstrated to safeguard the cardiovascular system by stabilizing cell membranes and reducing lipid peroxidation [15]. Enzymatic antioxidants such as superoxide dismutase (SOD) and catalase are crucial in deactivating superoxide radicals and hydrogen peroxide, both of which are increased in MI and lead to cellular damage [16]. Both SOD mimetics and catalase-based treatments have demonstrated potential in research models by boosting antioxidant protection and decreasing the size of heart attacks. Moreover, allopurinol, a xanthine oxidase inhibitor, has been examined for its capacity to decrease ROS generation and has demonstrated advantages in enhancing cardiac function in post-MI individuals [17]. Together, these treatments highlight how using antioxidants and enzymes can help reduce oxidative stress, prevent heart damage, and improve outcomes for patients with MI [18,19].

Therefore, this study aimed to investigate the mechanistic associations and clinical correlations of specific ROS and RNS markers in patients with MI compared to healthy controls. By simultaneously analyzing multiple individual reactive species and linking them with key biochemical and echocardiographic parameters, we sought to provide a more detailed understanding of oxidative and nitrosative stress dynamics in acute myocardial infarction. This mechanistic approach not only enhances our comprehension of MI pathophysiology but also supports the potential clinical utility of ROS and RNS as complementary biomarkers and future therapeutic targets.

## 2. Materials and Methods

### 2.1. Study Population

This study was carried out at the Medina Cardiac Center (MCC) in Al-Madinah Al-Munawara, Saudi Arabia. The study protocol was approved by the Research Ethics Committee, College of Medicine, Taibah University, Saudi Arabia (Approval code: PEP4-M8-V2; Approval date: 1 January 2020). This case–control study recruited a total of 86 MI patients and 60 subjects without a history of cardiovascular disease (CVD) as controls. The sample size was determined using power analysis (G*Power version 3.1.9.7, Heinrich-Heine-Universität Düsseldorf, Germany; http://www.gpower.hhu.de, accessed on 28 October 2020), based on preliminary differences in ROS/RNS levels between MI and control groups. With an assumed effect size (Cohen’s d) of 0.6, α = 0.05, and power (1 − β) = 0.80, a minimum of 50 subjects per group was required. Our final sample met and exceeded this threshold, ensuring sufficient power for meaningful comparisons. This study aimed to assess oxidative stress levels at the time of myocardial infarction (MI). Eligible participants were adult males and females aged 18 years or older who had been admitted with a diagnosis of either ST-elevation myocardial infarction (STEMI) or non-ST-elevation myocardial infarction (NSTEMI). Individuals were excluded if they had recent infections or chronic inflammatory conditions such as inflammatory bowel disease, hepatitis C, liver cirrhosis, malignancy, pregnancy, advanced liver or kidney disease, or chronic coronary syndrome. The control group comprised 60 apparently healthy individuals, matched by age and sex, with no documented history of cardiovascular disease, diabetes, or hypertension. Potential control participants were screened through clinical history, physical examination, blood pressure measurement, and laboratory investigations (including lipid profile and random blood glucose). Only those with normal findings and no use of medications affecting oxidative stress were included. This design ensured a metabolically healthy control group, offering a clear oxidative stress contrast with the MI cohort.

Informed consent was obtained from all participants. Identifying information was anonymized and treated with strict confidentiality. Demographic data and conventional CAD risk factors (e.g., dyslipidemia, diabetes, hypertension, obesity, smoking) were collected for both groups. MI patients were receiving standard acute coronary syndrome (ACS) therapies, including antiplatelets, antihypertensives, and antidiabetics, as clinically indicated. A full list of medications used by both groups is provided in Supplementary Table S1. Although red blood cell (RBC) values were initially analyzed as pooled data, we conducted a sex-stratified analysis given physiological differences in hematological norms between males and females. These findings are reported in Supplementary Table S2. While we acknowledge that variations in baseline medication use may affect oxidative stress parameters, the control group was intentionally composed of healthy individuals to serve as a reference standard. Routine investigations included serum lipids, complete blood count (CBC), random blood glucose, electrocardiogram (ECG), and echocardiography. Coronary angiography was performed on all MI patients.

### 2.2. Sample Collection and Analysis

#### 2.2.1. Sample Collection Procedures

Blood serum samples were collected from patients upon admission to the hospital, before any tests or treatments were conducted on either the MI or control groups. Serum samples were obtained from MI patients immediately upon hospital admission, before the administration of any acute-phase treatments. This approach was chosen to reduce potential interference from medications on oxidative marker levels. The samples were collected using sterile methods and then flushed with helium gas for 60 s at a flow rate of 1 mL/min to prevent free radicals from degrading. Next, the samples were centrifuged at 2000× *g* for 10 min at 4 °C. Subsequently, the samples were stored at −70 °C until analysis to maintain biomarker integrity. This cold storage minimizes the degradation of reactive species. The thawing process of the samples was performed slowly at 4 °C.

The analysis of the samples took place at the Taibah University College of Pharmacy in Al-Madinah Al-Munawara, Saudi Arabia. We analyzed the blood samples for oxidative markers like hydrogen peroxide (H_2_O_2_), peroxyl radical (ROO•), singlet oxygen (^1^O_2_), hydroxyl radical (•OH), hypochlorite radical (OHCl•), and superoxide anion (O_2_•^−^) to study reactive oxygen species (ROS), as well as nitric oxide (NO•), nitrogen dioxide (ONO-), and peroxynitrite (ONOO-) to examine reactive nitrogen species (RNS).

Fluorescence-based reagents were selectively utilized to measure ROS and RNS levels in the serum of both MI patients and control volunteers. Following the recommendations provided by Murphy et al. (2022) [20], we carried out our assessments of ROS and RNS to improve precision and replicability.

#### 2.2.2. Measurements Procedures for ROS

Hydrogen peroxide levels were measured using the Amplex Red Hydrogen Peroxide/Peroxidase Assay Kit (Molecular Probes, Eugene, OR, USA) [21]. Samples of serum were mixed with Amplex Red at a concentration of 10 mM and 1 U/mL HRP was incubated at room temperature for 5 min in 50 mM Tris buffer (pH 7.4). The resulting reaction mixture was then diluted threefold before fluorescence analysis. Assessment of the diluted Amplex Red solution’s fluorescence was performed using excitation at 563 nm and emission at 587 nm, in comparison to a freshly prepared H_2_O_2_ reagent solution [21].

Hydroxyl radical levels were assessed using coumarin-3-carboxylic acid (3-CCA) obtained from Sigma-Aldrich (Seelze, Germany). A 5 mM 3-CCA solution was prepared in 20 mM phosphate buffer (pH 7.4) at room temperature. Serum samples were incubated with the reagent for 2 min, after which fluorescence was measured at 450 nm following excitation at 395 nm. Results were compared to a standard curve generated using hydroxyl radicals produced by the Fenton reaction, which involved hydrogen peroxide and ferrous ammonium sulfate (Sigma-Aldrich) in phosphate buffer at pH 7.4 [22].

The quantity of superoxide anion was measured by determining the quantity of fluorogenic ethidium (E+) generated from the specific oxidation reaction of hydroethidine (HE) (Sigma-Aldrich) with the superoxide anion. The serum sample was mixed with a solution of 63.5 mM HE in DMSO and then diluted with a phosphate buffer at pH 7.8. The process of HE oxidizing to E+ was measured using fluorescence analysis, exciting at 470 nm and detecting emission at 590 nm, compared to a similarly treated standard. Potassium superoxide from Sigma-Aldrich was utilized as the standard reference for superoxide anion [23].

The Singlet Oxygen Sensor Green (SOSG) reagent from Molecular Probes in Eugene, Oregon, USA, was employed to detect singlet oxygen through fluorescence. The compound tetra-sulfonated porphine tetra (p-phenylsulfonate), also known as TPPS, was utilized to produce singlet oxygen as a standard for calibration in a study [24]. A volume of 20 µL of a 1 mM solution of SOSG prepared in methanol was mixed with 20 µL of the serum sample and incubated at 37 °C for 2 h in a dark chamber. Fluorescence emission was measured at 536 nm after excitation at 480 nm, and values were compared to a standard [24].

The APF (2-[6-(4′-amino) phenoxy-3H-xanthen-3-on-9-yl] benzoic acid) reagent from Sigma-Aldrich was used to selectively detect the hypochlorite radical, with fluorescence detection carried out and sodium hypochlorite from Sigma-Aldrich utilized for calibration [25]. Twenty microliters (20 μL) of 0.1 M sodium phosphate buffer (pH 7.4) and 20 μL of 0.25 M sodium hydrogen carbonate solution were combined with 2 μL of 10 μM APF solution in 1 mL sample tubes with 20 µL of serum sample, all shielded from light. The fluorescence intensity was observed using λex = 490 nm and λem = 520 nm.

Molecular Probes’ BODIPY581/591 lipid peroxidation sensor was utilized for peroxyl radical detection using fluorescence, with tert-butylhydroperoxide from Sigma-Aldrich for calibration [26]. A serum sample of 200 µL was combined with a solution including 100 µL of 20 µM BODIPY in PBS and 100 µL of PBS. Following 20 s of vertexing, the mixture was incubated at 37 °C under aerobic conditions for 10 min. The volume was subsequently adjusted to 1 mL using PBS. The amount of peroxyl radicals present was determined by measuring the increase in green fluorescence of the oxidation product using excitation and emission wavelengths of 500 nm and 520 nm.

#### 2.2.3. Measurements Procedures for RNS

DAF-2 was utilized as a selective fluorescent probe for the detection of nitric oxide (NO) radicals, with spermine NONOate serving as the NO donor. Serum samples were combined with 100 µL of sodium phosphate buffer and 100 µL of 10 mM DAF-2 dissolved in DMSO, and the mixture was incubated for approximately 1 min before analysis. Fluorescence was detected at 495 nm excitation and 515 nm emission wavelengths when compared to a standard that had been treated similarly [27].

A kit from Sigma-Aldrich, which utilizes 2,3-diaminonaphthalene (DAN) fluorescence, detected the presence of nitrite. In the dark, 150 µL of the serum sample was mixed with 75 µL of 158 µM DAN solution and 75 µL of 1.5 N HCl, then incubated at 30 °C for 5 min. Following this, 10 µL of 3.0 N NaOH was added, and fluorescence was measured using an excitation wavelength of 365 nm and emission at 410 nm. Results were compared against a standard curve generated using sodium nitrite [28].

To quantify peroxynitrite levels, nitrate reductase at a concentration of 5 U/mL was used to convert it into nitrite. DAN from Sigma-Aldrich was utilized to detect the resulting product [28].

### 2.3. Statistical Analysis

All statistical analyses were conducted using IBM SPSS Statistics version 20 (IBM Corp., Armonk, NY, USA). Continuous variables were initially tested for normality using the Shapiro–Wilk test, which revealed non-normal distribution for most variables. Therefore, non-parametric tests were employed for all between-group comparisons.

For comparisons between two independent groups (e.g., MI vs. control), the Mann–Whitney U test was used. For comparisons involving more than two groups (i.e., STEMI, NSTEMI, and control), the Kruskal–Wallis H test was applied. Where statistically significant differences were detected with the Kruskal–Wallis H test, followed by Dunn’s post hoc correction to adjust for multiple comparisons and reduce Type I error. This correction was used to maintain the overall family-wise error rate below 5%.

Correlations between oxidative/nitrosative stress markers and clinical or biochemical variables were assessed using the Spearman correlation test. The Evans’ classification [29] was used to interpret correlation strength: very strong (r > 0.80), strong (r = 0.60–0.79), moderate (r = 0.40–0.59), weak (r = 0.20–0.39), and very weak (r < 0.20). All statistical tests were two-tailed, and *p* < 0.05 was considered statistically significant unless otherwise adjusted.

## 3. Results

### 3.1. Demographic Data

This is a case–control study that recruited 146 subjects. We studied one group of MI patients (*N* = 86) versus control group (*N* = 60) (Table 1). There was no statistically significant difference between the two groups regarding age, gender, smoking status, and family history of coronary artery disease.

### 3.2. Hematological Tests and Lipid Profile

The systolic blood pressure (BP) was significantly higher in the MI group than in the controls, as shown in Table 2, median ± IQR; 125 (110–140) mmHg and 117 (110–125) mmHg, respectively (*p* = 0.001). The diastolic BP showed no significant difference between the MI cases and the controls. Moreover, the body mass index (BMI) of the cases did not show a significant statistical difference from the controls (*p* = 0.13). Random blood glucose showed a statistically significant difference between the cases and the controls. Median ± IQR was 151 (120–200) mg/dL in the cases and 117 (110–135) mg/dL in the controls (*p* = 0.004). There was a notable difference in the lipid parameters between the two groups (*p* < 0.001). Moreover, hematological parameters such as hemoglobin, white blood cell count, and platelet count, showed significant differences between the two groups (*p* < 0.001). Given the known sex-related differences in red blood cell counts, we performed a subgroup analysis stratified by sex. As shown in Supplementary Table S2, there were no statistically significant differences in RBC counts between MI patients and controls within each sex group, supporting the validity of our pooled analysis in the main text. Additionally, the median ± IQR of serum troponin of the cases was 15.5 (13.8–17.0) ng/mL, while the median ± IQR of left ventricular ejection fraction (LVEF) of the cases was 45 (40–50) %, respectively.

### 3.3. Reactive Oxygen and Nitrogen Species Serum Levels

As shown in Table 3 serum levels of reactive oxygen species (ROS) were significantly increased in MI patients compared to controls. The median ± IQR level of hydrogen peroxide was 531 (420–620) nM in subjects. patients with MI compared to 352 (280–450) nM in controls (*p* = 0.008). Hydroxyl radical levels were 217 (180–260) nM in patients with MI, while it was 154. (120–200) nM in the control group (*p* ≤ 0.001) similarly. Superoxide ion levels were significantly higher in MI patients, with a median ± IQR of 161 (120–200) nM vs. 103.5 (80–130) nM (*p* ≤ 0.001) in controls. Singlet oxygen levels were 135 (100–170) nM in MI patients versus 91.5 (70–120) nM in controls (*p* = 0.005). Hypochlorite radical levels were 62.5 (40–85) nM in MI patients and 36 (20–65) nM in controls (*p* = 0.01), while peroxyl radical levels were 74.5 (50–100) nM and 45.5 (30–70) nM, respectively (*p* = 0.002). With respect to reactive nitrogen species (RNS), nitric oxide levels were significantly higher in MI patients, with a median ± IQR of 12.80 (10–15) μM vs. 8.7 (6.5–11) μM (*p* ≤ 0.001) at the control level. Peroxynitrite was also increased in MI patients, with controls having a median ± IQR of 6.85 (6–7.5) μM vs. 4.95 (4–6) μM (*p* ≤ 0.001). However, nitrogen dioxide levels did not differ significantly between the two groups, with a median ± IQR of 6.45 (5.5–7.5) µM in MI patients and 6.15 (4.5–7.2) µM in controls (*p* = 0.315).

### 3.4. Correlation Between the ROS and RNS and Biochemical Markers/Echocardiographic Parameters

**Figure 1:**
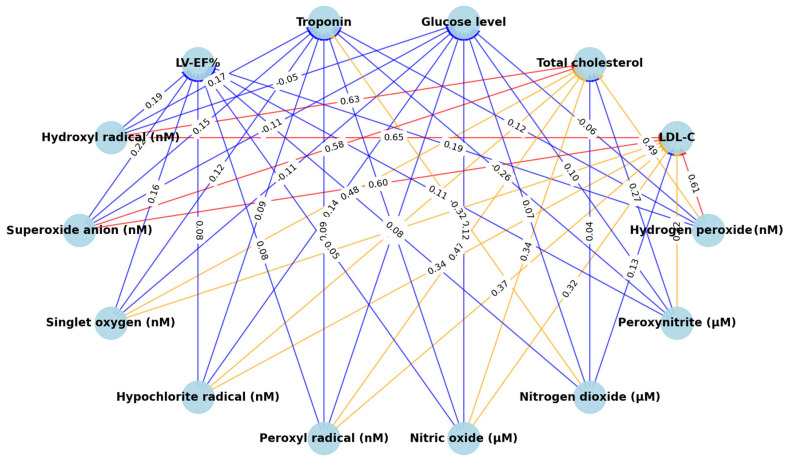
Network diagram illustrating correlations between reactive oxygen species (ROS), reactive nitrogen species (RNS), and key clinical markers in MI patients. Correlation strength is color-coded: Red = strong (r ≥ 0.6), Orange = moderate (0.4 ≤ r < 0.6), Blue = weak (0.2 ≤ r < 0.4). All correlations shown are statistically significant (*p* < 0.05).

**Figure 2:**
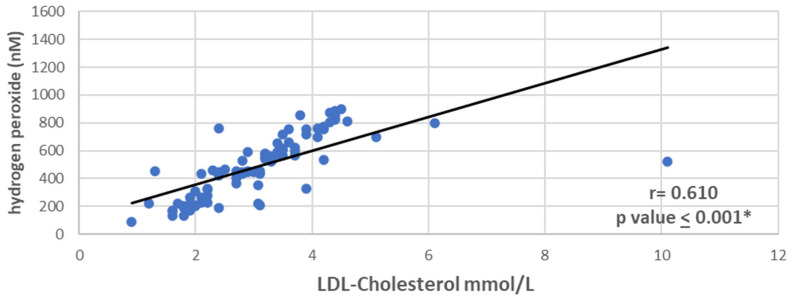
Scatter plot shows the correlation between hydrogen peroxide levels (a ROS marker) and LDL-cholesterol in MI patients. Spearman’s rank correlation was used due to non-normal data distribution. * *p* < 0.05 is considered significant. Abbreviations: MI. myocardial infarction; LDL-C, low density lipoprotein cholesterol.

Spearman’s coefficients (r) have been used to determine the relationships between biochemical markers and serum levels of ROS and RNS in MI. The results are shown in Table 4. Spearman’s correlation coefficients (r) were calculated to correlate the serum levels of ROS and RNS and different biochemical parameters in MI patients. Colorful network diagram illustrating the interactions between ROS and RNS, and key clinical markers (Figure 1). Significant positive associations between both se-rum LDL-cholesterol and total cholesterol and the ROS serum levels (*p* value < 0.001, highly significant). While LDL-cholesterol and total cholesterol showed a week positive relationship but still statistically significant *p*-value with nitrogen oxide and peroxynitrite. It is noted that nitrogen dioxide has no significant correlation with LDL cholesterol or total cholesterol (*p* value > 0.05). Serum glucose and troponin levels did not show a significant correlation with ROS or RNS, as *p* value is not significant except for nitrogen dioxide. Moreover, left ventricular ejection fraction does not show a significant correlation with all the studied serum levels of ROS or RNS. Data distribution with the correlation results between hydrogen peroxide (one of ROS) and LDL-cholesterol in cases with MI are presented in Figure 2.

The serum levels of various ROS and RNS were measured in myocardial infarction (MI) patients, categorized into STEMI (*n* = 62), NSTEMI (*n* = 24), and control (*n* = 60) groups.

**Figure 3:**
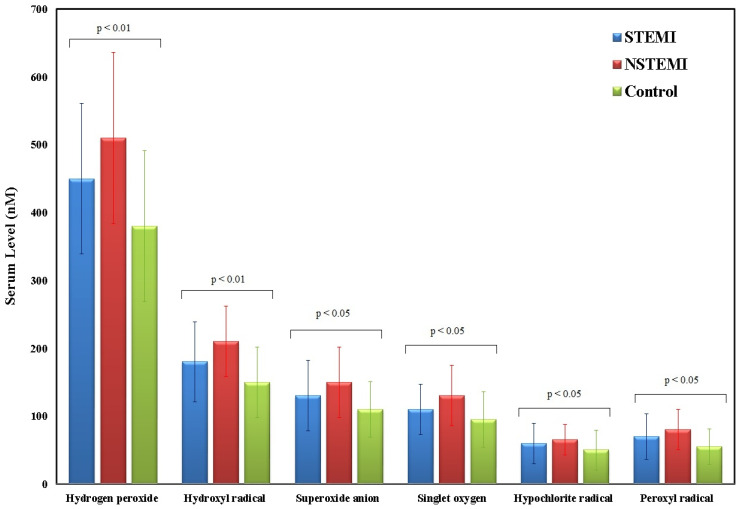
Serum levels of ROS including hydrogen peroxide, hydroxyl radical, superoxide anion, singlet oxygen, hypochlorite radical, and peroxyl radical among STEMI (*n* = 62), NSTEMI (*n* = 24), and control (*n* = 60) groups. Data presented as median and interquartile range (IQR). Kruskal–Wallis’s test followed by Dunn’s post hoc correction was used for comparisons. *p* < 0.05 indicates significance.

For ROS (Figure 3), STEMI patients showed hydrogen peroxide levels of 450 (380–530) (*p* < 0.01), hydroxyl radical at 180 (150–230) (*p* < 0.05), superoxide anion at 130 (100–170) (*p* < 0.01), singlet oxygen at 110 (90–140) (*p* < 0.05), hypochlorite radical at 60 (40–80) (*p* < 0.05), and peroxyl radical at 70 (50–95) (*p* < 0.05). NSTEMI patients showed higher levels in most ROS parameters, with hydrogen peroxide at 510 (430–600) (*p* < 0.001), hydroxyl radical at 210 (180–250) (*p* < 0.01), superoxide anion at 150 (120–190) (*p* < 0.01), singlet oxygen at 130 (100–160) (*p* < 0.05), hypochlorite radical at 65 (50–80) (*p* < 0.05), and peroxyl radical at 80 (60–100) (*p* < 0.05). In controls, levels were generally lower, with hydrogen peroxide at 380 (300–450), hydroxyl radical at 150 (120–190), superoxide anion at 110 (85–140), singlet oxygen at 95 (70–125), hypochlorite radical at 50 (30–70), and peroxyl radical at 55 (40–75). Statistical analysis was performed using the Kruskal–Wallis test, which revealed significant differences among the three groups for all ROS markers (*p* < 0.05). Dunn’s post hoc correction was applied for pairwise comparisons.

**Figure 4:**
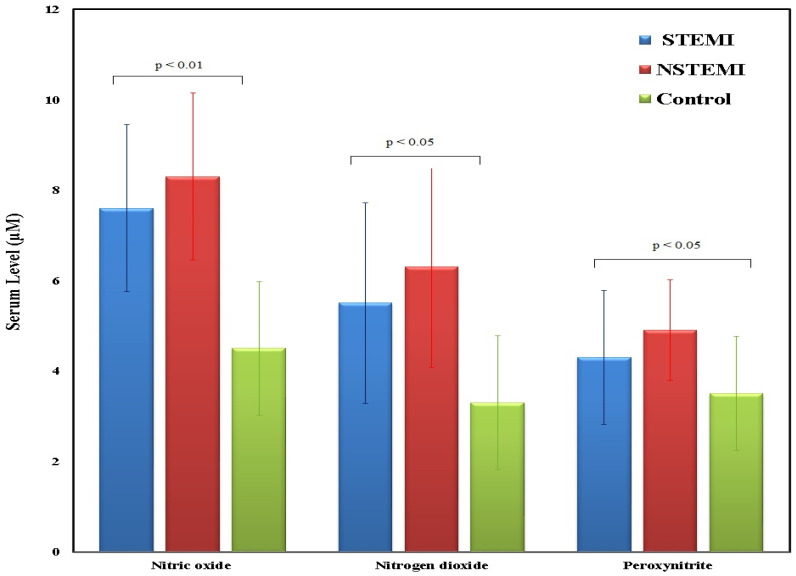
Serum levels of RNS including nitric oxide, nitrogen dioxide, and peroxynitrite in STEMI (*n* = 62), NSTEMI (*n* = 24), and control (*n* = 60) participants. Data are expressed as median (IQR). Kruskal–Walli’s test with Dunn’s correction was applied for multiple comparisons.

Figure 4 serum levels RNS, specifically nitric oxide, nitrogen dioxide, and peroxynitrite, among MI patients divided into STEMI (*n* = 62), NSTEMI (*n* = 24), and control (*n* = 60) groups. STEMI patients had nitric oxide levels of 7.6 (6.5–9.0) (*p* < 0.05), nitrogen dioxide at 5.5 (4.5–7.0) (*p* < 0.01), and peroxynitrite at 4.3 (3.5–5.5) (*p* < 0.01). STEMI patients exhibit intermediate RNS levels, higher than the control group but lower than STEMI patients. NSTEMI patients showed even higher levels, with nitric oxide at 8.3 (7.0–10.0) (*p* < 0.01), nitrogen dioxide at 6.3 (5.0–8.0) (*p* < 0.01), and peroxynitrite at 4.9 (4.0–6.0) (*p* < 0.05), while controls had nitric oxide at 4.5 (3.5–5.5), nitrogen dioxide at 3.3 (2.5–4.0), and peroxynitrite at 3.5 (2.8–4.5). NSTEMI patients demonstrate the highest serum levels for all three RNS markers, with a broad distribution and elevated medians compared to both NSTEMI and control groups. In contrast, the control group consistently shows the lowest median RNS levels and the smallest interquartile ranges. These results indicate that RNS levels are significantly elevated in MI patients, particularly those with STEMI, suggesting a potential association between increased RNS and the severity of myocardial infarction. Statistical analysis using the Kruskal–Wallis’s test indicated significant group-wise differences for all three markers. Dunn’s post hoc correction was subsequently applied for pairwise comparisons.

## 4. Discussion

The development and progression of MI are influenced by various factors, with emerging research highlighting the importance of ROS and RNS [8]. These chemicals are highly reactive and can cause oxidative stress and inflammation. ROS and RNS are crucial in the growth and advancement of MI [11,13]. Excessive ROS production can occur during conditions like hypertension, hyperlipidemia, diabetes mellitus and myocardial infarction [30]. Taniyama et al. [31] found that NAD(P)H oxidases, xanthine oxidase (XO), and myeloperoxidase (MPO) are potential sources of superoxide. Moreover, Barry-Lane et al. [32] showed that the production of ROS by NAD(P)H oxidase plays a crucial role in the development of atherosclerotic lesions and subsequent cardiovascular disease (CVD). This could impact clinical practice, and new research is looking for scavengers of ROS to promote myocardial healing post-MI [33].

To the best of our knowledge, the association between the development of MI, i.e., STEMI and NSTEMI, and the ROS and RNS markers had yet to be studied in detail before this study. While oxidative stress has been implicated in cardiovascular diseases, its precise role in predicting long-term outcomes following MI remains unclear. Determining whether ROS/RNS are predictive, mechanistically involved, or merely epiphenomena would require prospective longitudinal studies. However, understanding these oxidative stress dynamics could provide insights for future research on whether ROS/RNS markers can serve as predictors of recurrent cardiovascular events, heart failure, or mortality. Although comorbidities such as diabetes and hypertension are known to influence oxidative stress, they were not adjusted for in this analysis because they were intentionally excluded from the control group to provide a clear contrast with the clinical profile of MI patients [34]. Smoking status did not differ significantly between groups (*p* = 0.14), minimizing its potential as a confounding factor. The inclusion of comorbidities in the MI group reflects the typical patient population and captures the real-world oxidative stress burden associated with myocardial infarction.

The biochemical markers were correlated with the levels of ROS and RNS, and the strongest correlations were for the hydroxyl and superoxide radicals, suggesting that they play a dominant role in the etiopathogenesis of atherosclerosis and, eventually, the development of myocardial infarction. Serum troponin showed weak correlations with ROS and RNS. Although troponins are a marker of myocardial injury [35], they are not associated with oxidant stress. Pathophysiological mechanisms have been postulated, e.g., neurohormonal activation and adrenergic activated cellular and mitochondrial calcium overload [36]. Prior studies have suggested that excessive ROS/RNS production contributes to post-MI complications, yet few studies have explored whether these oxidative markers have long-term prognostic significance. If validated through future cohort studies, oxidative stress biomarkers could be integrated into cardiovascular risk models to refine patient stratification and treatment approaches.

LDL levels strongly correlated with hydrogen peroxide radicals, although the correlation was weak or very weak for the other ROS. Regarding the RNS markers, the analysis showed a negative correlation. LDL cholesterol is a well-known risk factor for the development of atherosclerotic CVD and is used routinely to assess the risk of the development of CVD [37]. In addition, Zhao and colleagues conducted a single observational study and showed that oxidized LDL was independently associated with the severity of coronary artery disease [38]. Moreover, total cholesterol strongly correlates with ROS markers, i.e., hydrogen peroxide and singlet oxygen, and has a moderate positive correlation with hydroxyl radicals. In contrast, RNS markers showed a negative correlation. In agreement with our results, a study on patients with CAD found that lipid profiles were significantly different in patients with coronary artery disease compared to the controls; moreover, they found a high level of plasma malondialdehyde (MDA), which is a product of lipid peroxidation [39]. On the contrary, HDL and triglycerides showed weak positive for ROS and negative correlations with RNS markers. It is well known that HDL-cholesterol has anti-inflammatory and anti-atherosclerotic properties. Interestingly, once the high oxidative stress oxidizes HDL-cholesterol, it promotes inflammation and atherosclerosis. Rho et al. studied patients with rheumatoid arthritis and found increased levels of urinary F2-isoprostane excretion; a marker of oxidative stress and serum HDL-cholesterol correlated with coronary artery calcification [40].

The variation in hemoglobin levels between the MI patients and the control group can be ascribed to the different health characteristics of the two populations. The MI group mainly included individuals exhibiting acute coronary syndromes, where systemic inflammatory responses and compensatory erythropoietic mechanisms frequently result in comparatively elevated hemoglobin levels [41]. In contrast, the control group was chosen for the lack of CVD but might still consist of people with undiagnosed or subclinical issues, such as anemia, stemming from age-related changes or other underlying causes.

In our analysis, we found that 55% of our patients with CAD also have diabetes mellitus. Thus, chronic hyperglycemia could induce oxidative stress and further progress to the development of atherosclerosis. A previous study on diabetic patients found an increase in NAD(P)H oxidase activity and uncoupling of eNOS as compared to matched control groups, which supports our findings [42].

Hypertension is a significant risk factor for the development of CVD [43]. T cell activation, with subsequent overproduction of IL-6, interferon-Ɣ, and tumor necrosis factor-α, plays a vital role in the pathogenesis of the disease [44]. T cells increase vascular ROS and segment renal angiotensin II production, which increases the production of ROS by activating the NADPH oxidase [45]. Several studies have confirmed that oxidative stress is linked to chronic hyperglycemia and obesity [46,47]. Nutritional overload increases blood glucose levels and free fatty acids. Excessive amounts of nutritional substrate are available to the cellular metabolic pathways, enhancing ROS production [48]. Oxidative stress mediated by the overproduction of ROS results in mitochondrial dysfunction and activates the NADPH oxidase enzyme, eventually causing myocardial injury [49].

It is stated that oxidative stress produced by increased ROS in the myocardium is pivotal in developing myocardial infarction and heart failure [50,51]. Moreover, Borchi et al. found that there are high levels of ROS mainly from the activity of NADPH oxidase in explanted failing hearts, primarily the right ventricle [52]. Moreover, the inhibition of ROS production has been shown to ameliorate post-myocardial infarction heart failure in mice through the activation of mitochondrial inner membrane uncoupling protein isoform [53]. The lack of a significant difference in nitrogen dioxide (NO_2_^−^) levels between MI patients and controls may suggest that this biomarker is less sensitive or specific for MI compared to other reactive nitrogen species. Unlike peroxynitrite and nitric oxide, which are more directly involved in oxidative stress and endothelial dysfunction in MI, nitrogen dioxide may be more influenced by external factors such as baseline inflammatory status. Additionally, its rapid conversion to other nitrogen species could contribute to variability in its serum levels.

In the MI research, it is crucial to differentiate between STEMI and NSTEMI due to their varying pathophysiological mechanisms and impact on patient care. STEMI is typically associated with a complete and prolonged blockage of a coronary artery, causing more myocardial necrosis, while NSTEMI is caused by a partial or temporary obstruction that can lead to less severe but continuous ischemic damage [6]. These variations in ischemic load might affect oxidative stress levels, with STEMI potentially causing acute spikes in ROS and RNS production owing to abrupt, intense oxygen deficiency, followed by reperfusion injury when blood supply is reinstated. Conversely, NSTEMI can be associated with persistent oxidative stress characterized by a continuous, though lesser, rise in ROS and RNS markers resulting from ongoing partial blockage and ischemia [9].

The different levels of ROS and RNS found in our STEMI and NSTEMI patients could indicate these unique mechanisms, possibly affecting the approach to managing oxidative stress in these groups. NSTEMI instances consistently display heightened baseline ROS and RNS, suggesting a potential requirement for focused antioxidant approaches in chronic management. In cases of STEMI, attention may shift to managing sudden oxidative damage after reperfusion [54]. The observed differences underscore the importance of tailoring treatments based on oxidative stress profiles in STEMI and NSTEMI to improve clinical results and minimize heart muscle injury.

Several studies have suggested that oxidative stress persists beyond the acute phase of MI and may contribute to long-term cardiac remodeling, endothelial dysfunction, and disease progression [18,19]. In the current study, we compared these oxidative stress markers across MI subtypes within a Saudi population, which remains underrepresented in the oxidative stress literature despite a high burden of cardiovascular disease. We also correlated ROS and RNS levels with key clinical biomarkers, including LDL cholesterol, total cholesterol, glucose, and troponin, to provide a more comprehensive understanding of the biochemical and pathophysiological milieu of acute MI. Importantly, all samples were collected in a cross-sectional case–control design, thereby reducing potential confounding from therapeutic interventions and strengthening the biological relevance of our findings.

The findings of this study suggest that ROS and RNS markers could have potential clinical applications in MI management, particularly in risk stratification, early detection, and patient monitoring. For instance, patients presenting with elevated oxidative stress biomarkers at admission could be flagged as high-risk individuals for adverse cardiovascular outcomes, thereby prompting more aggressive early intervention or closer monitoring during hospitalization. Additionally, these biomarkers could help differentiate between unstable angina and MI in borderline cases, especially when troponin levels are equivocal. Serial measurements of ROS/RNS might also serve as a tool to monitor therapeutic response, particularly to antioxidant-based therapies or revascularization procedures. Furthermore, distinct oxidative stress profiles in STEMI vs. NSTEMI could inform tailored antioxidant strategies, such as targeting reperfusion injury in STEMI or chronic oxidative burden in NSTEMI. If validated in larger cohorts, these markers could be integrated into multi-biomarker panels to complement traditional risk models, ultimately supporting personalized treatment pathways and improving clinical decision-making.

However, our study only provides a snapshot of oxidative stress levels at the time of MI, without follow-up data to assess whether these markers correlate with patient outcomes. Moving forward, a cohort study tracking oxidative stress levels at multiple time points post-MI could help establish their role as independent prognostic indicators. Additionally, investigating whether antioxidant therapies can mitigate oxidative damage and improve cardiovascular outcomes remains a crucial area for future exploration.

## 5. Conclusions

This study is among the first to comprehensively analyze individual reactive oxygen and nitrogen species (ROS and RNS) in patients with myocardial infarction (MI), providing detailed mechanistic insights beyond the assessment of overall oxidative stress. Our findings demonstrate significant elevations in multiple ROS and RNS markers among MI patients compared to healthy controls, with distinct patterns observed in both STEMI and NSTEMI subtypes. While correlations with troponins, glucose levels, and left ventricular ejection fraction were weak or non-significant, moderate to strong associations with total cholesterol and LDL-cholesterol suggest a mechanistic link between lipid metabolism and oxidative stress in acute MI. These results highlight the potential of ROS and RNS markers as complementary biomarkers for assessing disease severity and the oxidative burden in MI. Moreover, understanding these oxidative and nitrosative stress dynamics may inform future therapeutic strategies aimed at targeting redox pathways to improve clinical outcomes in MI patients.

## Figures and Tables

**Table 1:** General characteristics of MI patients and control subjects.

Variables	Participants (n = 146)		*p*-Value *^a^*
	MI Patients (n = 86) (%)	Control (n = 60) (%)	
Age:			0.48 (NS)
Median	57.0	53.0	
IQR	15.5	10.5	
Gender:			0.49 (NS)
Male	66 (76.7%)	43 (71.7%)	
Female	20 (23.3%)	17 (28.3%)	
Smoking:			0.14 (NS)
No	50 (58.1%)	42 (70%)	
Yes	36 (41.9%)	18 (30%)	
Family History of CAD:			0.90 (NS)
No	71 (82.1%)	50(83.3%)	
Yes	15 (17.9%)	10 (16.7%)	
History of DM:			
No	39 (45.3%)	0 (0%)	N/A
Yes	47 (54.7%)	0 (0%)	N/A
History of hypertension:			
No	37 (43%)	0 (0%)	N/A
Yes	49 (57%)	0 (0%)	N/A

*^a^* Chi square test. *p*-value < 0.05 is considered statistically significant. Abbreviations: MI, myocardial infarction; CAD, coronary artery disease; DM, diabetes mellitus; IQR, interquartile range; N/A, nonapplicable.

**Table 2:** Hematological parameters, lipid profile, and key clinical measurements in MI patients and control subjects.

Parameters	MI Patients (*n* = 86) Median ± IQR	Control (*n* = 60) Median ± IQR	*p*-Value *
Systolic blood pressure (mmHg)	125 (110–140)	117 (110–125)	0.001 (HS)
Diastolic blood pressure (mmHg)	73 (68–80)	75 (70–80)	0.62 (NS)
BMI	28 (25–32)	27 (24–30)	0.13 (NS)
Glucose Level, random (mg/dL)	151 (120–200)	117 (110–135)	0.004 (S)
Total cholesterol (mmol/L)	5 (4.2–6)	3.04 (2.8–3.5)	<0.001(HS)
LDL-cholesterol (mmol/L)	3 (2.2–3.8)	2 (1.6–2.4)	<0.001 (HS)
HDL-cholesterol (mmol/L)	1 (0.9–1.1)	1.16 (1.1–1.2)	<0.001 (HS)
Triglycerides (mmol/L)	1 (0.8–1.8)	1.04 (0.8–1.4)	0.04 (S)
Hb (g/dL)	14 (12.5–15.7)	11 (10.7–11.9)	<0.001 (HS)
RBCs (10^6^/uL)	5 (4.5–5.6)	4.8 (4.4–5.1)	0.08 (NS)
Total WBCs (10^3^/uL)	10 (8–13)	7.3 (6.4–8)	<0.001 (HS)
Platelets (103/uL)	262 (220–310)	212.5 (200–230)	<0.001 (HS)
Troponin (ng/mL)	15.72 + 4.2 (15.5)	-	N/A
LV-EF (%)	45 (40–50)	-	N/A

* Shapiro-Wilk test showed that the data are not normally distributed. So, *p*-values were test using Mann–Whitney U test. *p*-value < 0.05 is considered statistically significant. Data are expressed as Median ± IQR. Abbreviations: BMI, body mass index; Hb, haemoglobin; HDL-C, high density lipoprotein cholesterol; HS, highly significant; LDL-C, low density lipoprotein cholesterol; LV-EF, left ventricular ejection fraction; NS, non-significant; RBCs, red blood cells; S, significant; IQR, interquartile range; WBCs, white blood cells, N/A, nonapplicable.

**Table 3:** Serum levels of reactive oxygen and nitrogen species in MI patients and control subjects.

	MI Patients (*n* = 86) Median ± IQR	Control (*n* = 60) Median ± IQR	*p*-Value *
Reactive oxygen species			
Hydrogen peroxide (nM)	531 (420–620)	352 (280–450)	0.008 (S)
Hydroxyl radical (nM)	217 (180–260)	154 (120–200)	≤0.001 (HS)
Superoxide anion (nM)	161 (120–200)	103.5 (80–130)	≤0.001(HS)
Singlet oxygen (nM)	135 (100–170)	91.5 (70–120)	0.005 (S)
Hypochlorite radical (nM)	62.5 (40–85)	36 (20–65)	0.01 (S)
Peroxyl radical (nM)	74.5 (50–100)	45.5 (30–70)	0.002 (S)
Reactive nitrogen species			
Nitric oxide (µM)	12.80 (10–15)	8.7 (6.5–11)	≤0.001 (HS)
Nitrogen dioxide(µM)	6.45 (5.5–7.5)	6.15 (4.5–7.2)	0.315(NS)
Peroxynitrite (µM)	6.85 (6–7.5)	4.95 (4–6)	≤0.001 (HS)

* As the data are not normally distributed (tested by the Shapiro–Wilk test), they are presented as Median ± IQR and *p*-value was calculated using Mann–Whitney U Test. *p*-value below 0.05 is considered significant and was bolded. Abbreviations: IQR, interquartile range; MI, myocardial infarction.

**Table 4:** Spearman’s correlation coefficients between serum ROS/RNS levels and clinical/biochemical markers in MI patients.

Reactive Species	LDL-C			Total Cholesterol	Glucose Level (Random)		Troponin		LV-EF%	
	r	*p*-Value	r	*p*-Value	r	*p*-Value	r	*p*-Value	r	*p*-Value
Reactive oxygen species										
Hydrogen peroxide (nM)	0.610	<0.001(HS)	0.492	<0.001(HS)	−0.06	0.48(NS)	0.121	0.27(NS)	0.19	0.08(NS)
Hydroxyl radical (nM)	0.647	<0.001(HS)	0.627	<0.001(HS)	−0.05	0.49(NS)	0.169	0.12(NS)	0.19	0.07(NS)
Superoxide anion (nM)	0.602	<0.001(HS)	0.576	<0.001(HS)	−0.110	0.19(NS)	0.146	0.18(NS)	0.22	0.05(S)
Singlet oxygen (nM)	0.444	<0.001(HS)	0.484	<0.001(HS)	−0.111	0.18(NS)	0.121	0.27(NS)	0.16	0.14(NS)
Hypochlorite radical (nM)	0.343	<0.001(HS)	0.425	<0.001(HS)	−0.14	0.08(NS)	0.09	0.37(NS)	0.08	0.46(NS)
Peroxyl radical (nM)	0.371	<0.001(HS)	0.465	<0.001(HS)	−0.10	0.21(NS)	0.09	0.37(NS)	0.08	0.44(NS)
Reactive nitrogen species										
Nitric oxide (µM)	0.321	<0.001(HS)	0.337	<0.001(HS)	0.123	0.18(NS)	−0.259	0.05(S)	0.15	0.73(NS)
Nitrogen dioxide(µM)	0.134	0.148(NS)	0.045	0.626(NS)	0.068	0.46(NS)	−0.322	0.01(S)	0.08	0.52(NS)
Peroxynitrite (µM)	0.320	<0.001(HS)	0.270	0.003(S)	0.10	0.27(NS)	−0.261	0.05(S)	0.11	0.42(NS)

Notes: As the data are not normaliy distrbuted (tested by Shapiro–Wilk test), corrleation coefficents were calculated by Spearman test. *p* value below 0.05 is conisdered signficant. MI, myocardial infarction; LDL-C, low density lipoprotein cholesterol; LV-EF, left ventrcular ejection fraction.

## Data Availability

The original contributions presented in this study are included in the article and its Supplementary Materials. All datasets generated and analyzed during the current study are available from the corresponding author upon reasonable request.

## Supplementary Materials

The following supporting information can be downloaded at: https://www.mdpi.com/article/10.3390/medsci13030152/s1, Table S1: The medication used in both the MI and control groups; Table S2: Red blood cell (RBC) count (×10^6^/μL) stratified by sex in MI patients and controls.
