# Supplementary material for: Reactive Oxygen and Nitrogen Species in Myocardial Infarction: Mechanistic Insights and Clinical Correlations
Source: Med Sci (Basel). 2025 Aug 24;13(3):152. doi: 10.3390/medsci13030152 (PMC12452750; doi:10.3390/medsci13030152)
Supplement: Supplementary file 1 [file medsci-13-00152-s001.zip › medsci-3756808-supplementary.pdf]

Table S1. The medication used in both the MI and control groups

Medication Class	MI Group (n=86) (%)	Control Group (n=60) (%)
Antiplatelets (Aspirin, Clopidogrel)	62.8 %	26.7%
Statins (Atorvastatin, Rosuvastatin)	55.7 %	53.3%
Anticoagulants (Heparin)	17.4 %	N/A
ACE Inhibitors / ARBs	48.8 %	N/A
Calcium Channel Blockers	38.4 %	N/A
Beta-blockers	52.3 %	N/A
Metformin / Insulin	13.9 %	N/A

Table S2. Red blood cell (RBC) count ($\times 10^6/\mu L$) stratified by sex in MI patients and controls.

Sex	MI Patients		Control Subjects		P-value
	n	RBC Count (Median ± IQR)	n	RBC Count (Median ± IQR)	
**Male**	66	5.1 (4.7–5.8)	43	4.9 (4.6–5.2)	0.07 (NS)
**Female**	20	4.5 (4.2–5.0)	17	4.4 (4.0–4.7)	0.18 (NS)

Data are expressed as Median ± IQR and p-value was calculated using Mann-Whitney U Test

Abbreviations: MI, myocardial infarction; **RBC**, red blood cell count; **NS**, non-significant.
